# Ultrasound-guided superior laryngeal nerve block assists in anesthesia for bronchoscopic surgical procedure: A case report of anesthesia for rigid bronchoscopy

**DOI:** 10.1097/MD.0000000000020916

**Published:** 2020-07-02

**Authors:** Yu-Chen Liao, Wei-Ciao Wu, Ming-Hui Hsieh, Chuen-Chau Chang, Hsiao-Chien Tsai

**Affiliations:** a Department of Anesthesiology; b Division of Thoracic Surgery, Department of Surgery, Taipei Medical University Hospital; c Department of Anesthesiology, School of Medicine, College of Medicine, Taipei Medical University; d Anesthesiology and Health Policy Research Center, Taipei Medical University Hospital; e Graduate Institute of Medical Sciences, College of Medicine, Taipei Medical University, Taipei, Taiwan.

**Keywords:** bronchoscopic surgical procedure, case reports, intravenous anesthesia, superior laryngeal nerve block

## Abstract

**Introduction:**

Evolving techniques in the field of therapeutic bronchoscopy have led to the return of rigid bronchoscopy in the treatment of complex central airway disease. Rigid bronchoscopy is typically performed under general anesthesia because of the strong stimulation caused by metal instruments. Anesthesia for rigid bronchoscopy is challenging to administer because anesthesiologists and interventionists share the same working channel: the airway. Previously reviewed anesthetic methods are used primarily for short procedures. Balanced anesthesia with ultrasound-guided superior laryngeal nerve (SLN) block and total intravenous anesthesia might provide anesthesia for a prolonged procedure and facilitate patient recovery.

**Patient concerns:**

A patient with obstructed endobronchial stent was referred for therapeutic rigid bronchoscopy, which requires deeper anesthesia than flexible bronchoscopy. There were concerns of the stronger stimulation of the rigid bronchoscopy, lengthy duration of the procedure, higher risk of hypoxemia, and the difficulty of mechanical ventilation weaning after anesthesia due to the patients co-morbidities.

**Diagnosis:**

A 66-year-old female patient presented with a history of breast cancer with lung metastases. Right main bronchus obstruction due to external compression of lung metastases was relieved through insertion of an endobronchial stent, but obstructive granulation developed after 4 months. Presence of the malfunctioning stent caused severe cough and discomfort. Removal of the stent by using a flexible bronchoscope was attempted twice but failed.

**Interventions:**

Regional anesthesia of the upper airway through ultrasound-guided SLN block combined with intratracheal 2% lidocaine spray was performed to assist in total intravenous anesthesia (TIVA) during rigid bronchoscopy.

**Outcomes:**

The patient maintained steady spontaneous breathing throughout the procedure without laryngospasm, bucking, or desaturation. Emergence from anesthesia was smooth and rapid after propofol infusion was discontinued. The surgery lasted 2.5 hours without discontinuity, and no perioperative pulmonary or cardiovascular complications were noted.

**Conclusion:**

Ultrasound-guided SLN block is a simple technique with a high success rate and low complication rate. Application of SLN block to assist TIVA provides sufficient anesthesia for lengthened therapeutic rigid bronchoscopy without interruption and facilitates patient recovery.

## 1 Introduction

The application of advanced therapeutic bronchoscopy is a rapidly increasing. It plays valuable role in the treatment of complex central airway disease, lung masses, and hilar adenopathy and offers minimally invasive procedures for both curative and palliative indications.^[1,2]^ A rigid bronchoscope is a metal tube with 3 ports for an optical device, a working instrument, and ventilation. Rigid bronchoscopes cause stronger laryngeal stimulation than do flexible bronchoscopes and are associated with arrhythmias, myocardial ischemia, and stroke.^[3]^ Considerations of anesthesia for rigid bronchoscopy include patient condition, procedure, anesthetic method, airway management, and ventilation technique. Anesthesia for rigid bronchoscopy usually requires general anesthesia, largely maintained with total intravenous anesthesia (TIVA).^[3,4]^ TIVA describes the technique to maintain anesthesia without inhaled anesthetics,^[5]^ which cannot be steadily delivered during anesthesia for bronchoscopy due to unstable ventilation. Ventilation is primarily provided through the side port of a rigid bronchoscope. Sharing airway access with interventionists during a procedure is associated with risk of hypoventilation, hypoxemia, desaturation, hypercapnia, and cardiovascular complications.^[3,6]^

Several ventilation techniques for rigid bronchoscopy have been reported, including apneic oxygenation, spontaneous assisted ventilation, controlled ventilation, and jet ventilation.^[3,6–9]^ Maintenance of adequate ventilation and oxygenation without adverse events during rigid bronchoscopy is a challenge. Furthermore, intermittent interruption of a procedure may be performed to prevent pulmonary and cardiovascular complications during a prolonged procedure.

**Figure 1:**
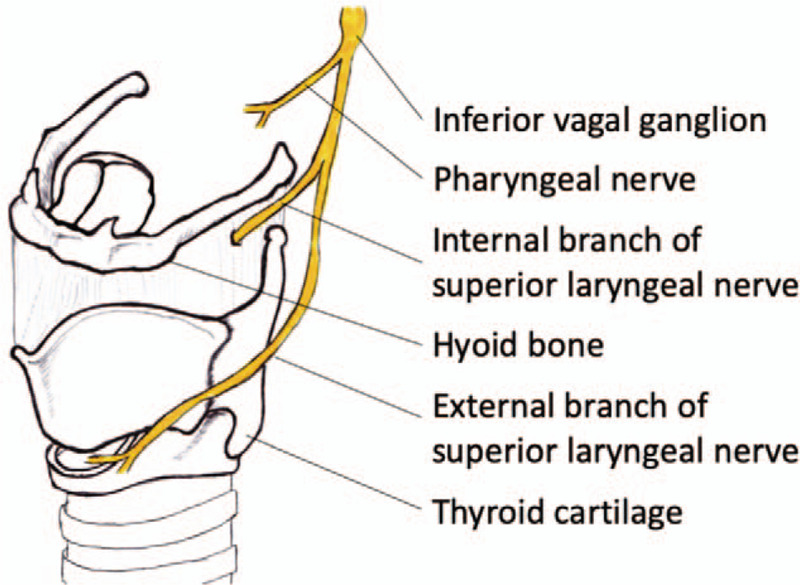
Anatomy of the superior laryngeal nerve. The superior laryngeal nerve (SLN) originates from the inferior vagal ganglion and diverges into an internal branch and an external branch. The internal branch of the SLN runs beneath or lateral to the greater horn of the hyoid and penetrates the thyrohyoid membrane to innervate the sensory system of the larynx.

The superior laryngeal nerve (SLN) innervates the sensation of the larynx (Fig. 1).^[10–13]^ SLN block has been applied to facilitate awake endotracheal tube intubation,^[11]^ endoscopic laryngeal surgery,^[14]^ and laryngeal surgery on patients with neuromuscular disorder.^[15]^ We assumed that SLN block might reduce airway irritation and inhibit laryngeal reflex during rigid bronchoscopy, thereby reducing the requirement for plasma concentration (Cp) of propofol and associated respiratory depression. We applied balanced anesthesia with ultrasound-guided SLN block and TIVA in a patient undergoing palliative therapeutic rigid bronchoscopy with a lengthy expected procedure time.

## 2 Case presentation

**Figure 2:**
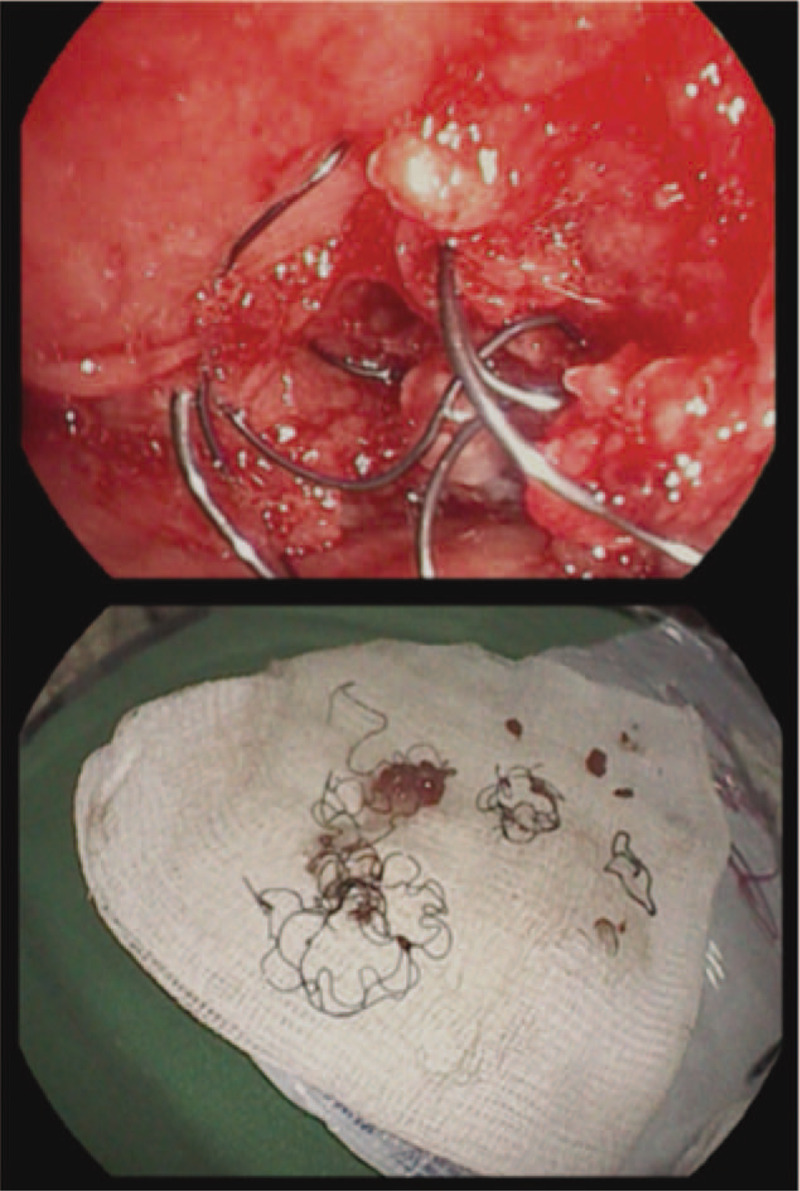
Flexible bronchoscopy was performed to remove the obstructed endobronchial stent, but it failed. (a) The distorted, crushed stent in the right main bronchus; (b) Only partially crushed fragments were removed through flexible bronchoscopy.

A 66-year-old Asian female patient presented with a history of breast cancer with lung metastasis that caused external compression of the right main bronchus and subsequent atelectasis of the right upper lung and dyspnea. An endobronchial stent was inserted for palliative symptom relief, but obstructive granulation occurred 4 months later. The presence of the functionless stent resulted in severe cough and dyspnea. The patient strongly requested removal of the stent. A chest physician twice attempted to remove the stent with cryotherapy and forceps, but only some fragments of the stent were removed (Fig. 2). The patient was referred for therapeutic rigid bronchoscopy to remove the residual endobronchial stent. Patient has provided informed consent for publication of the case.

Upon arrival at the operation room, the patients vital signs were recorded as follows: oxygen saturation, 92% to 95% under nasal cannula 3L/minute; blood pressure, 111/73 mm Hg; and heart rate, 86 bpm. Physical examination revealed a cachexic appearance and the attenuation of breathing sounds in the right upper lung field. In consideration of the difficulty of mechanical ventilation weaning and the palliative status of the patient, we planned to anesthetize the patient through intravenous anesthesia in combination with regional anesthesia of the airway. We expected to maintain adequate spontaneous breathing while minimizing airway irritation and adverse events during the procedure.

**Figure 3:**
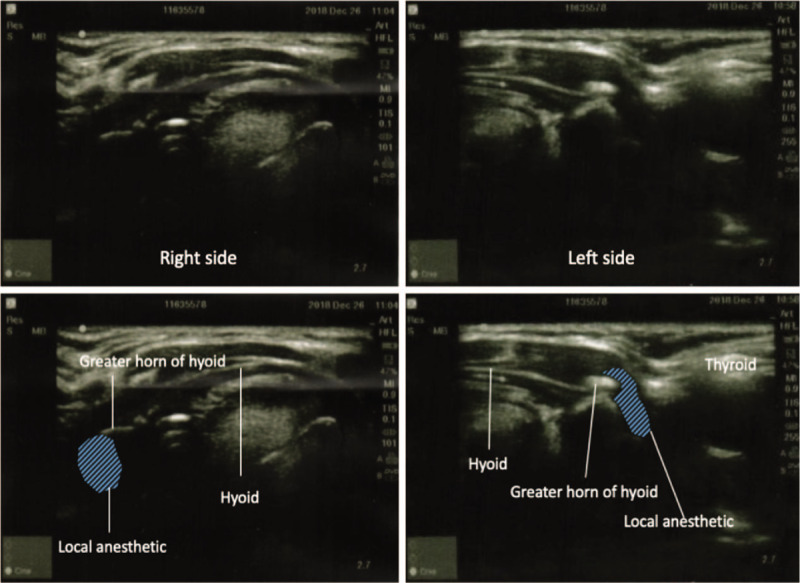
Ultrasound-guided superior laryngeal nerve block performed through a transverse approach. A 1.5-ml injection of 2% lidocaine was performed around the greater horn of the hyoid bone, which was identified as the sonographic landmark.

**Figure 4:**
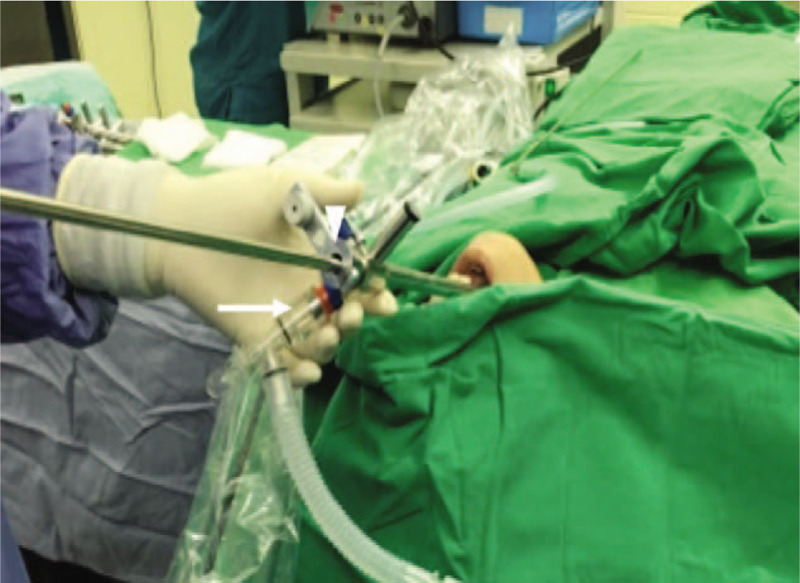
A rigid bronchoscope with a large metal working channel was inserted and occupied the larynx and airway, causing considerable stimulation throughout the procedure. Oxygen could only be supplemented through the side port of the bronchoscope and leaked from the working channel during rigid bronchoscopy. White arrow: side port for oxygen supplement; white triangle: circuit leakage through the working channel.

**Figure 5:**
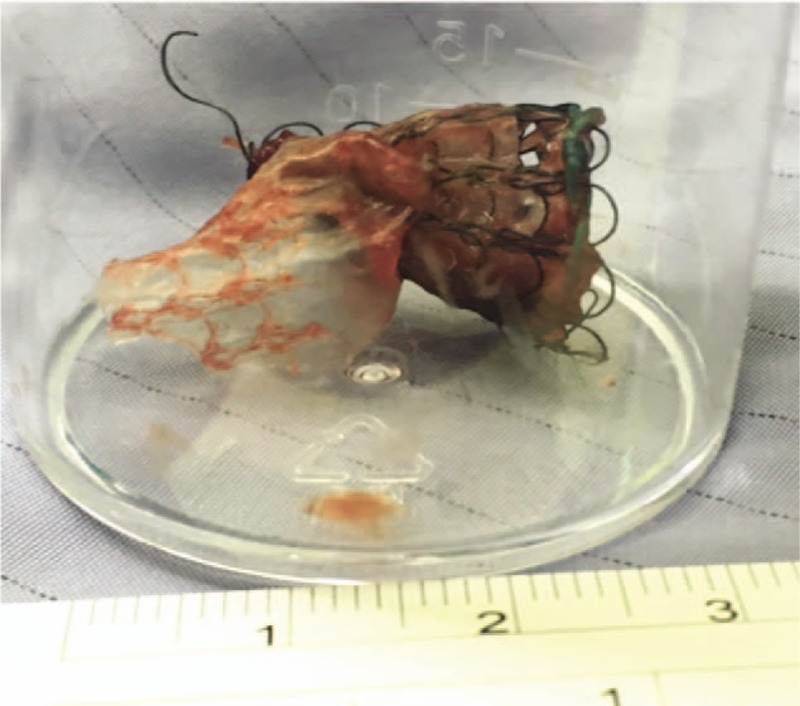
A residual bronchial stent with dimensions of 2.5 cm × 1 cm × 1 cm was successfully removed through rigid bronchoscopy.

Under standard monitoring, mild sedation was induced with 1 mg of midazolam and 50 μg of fentanyl. Target-controlled infusion (TCI) of propofol was applied with the Marsh model (Cp titrated between 1.8 and 2.6 μg/ml) to achieve hypnosis while preserving steady spontaneous breathing. To minimize airway secretion and salivation, 0.2 mg of glycopyrrolate was administered. The patient was placed in a supine position with neck extension. Transverse-approach ultrasound-guided SLN block was performed bilaterally using a SonoSite HFL38 linear probe (13–6 MHz) (Fig. 3). The greater horn of the hyoid was identified as a sonographic landmark under the transverse view of the neck, and 1.5 ml of 2% lidocaine was injected with a 25-gauge needle to infiltrate around it. Furthermore, an intratracheal spray with 6 ml of 2% lidocaine was applied using a flexible bronchoscope to block sensation from the vocal cord to the right main bronchus lesion. A large metal rigid bronchoscope was maintained in the trachea throughout the procedure without bucking, laryngospasm, or bronchospasm (Fig. 4). Pure oxygen was supplied through the side port of the rigid bronchoscope. The patient manifested steady spontaneous breathing without desaturation, and the hemodynamic condition was stable throughout the 2.5-hour procedure time. Oxygen saturation was maintained at 97% to 100% during the intraoperative period. There was neither interruption of the procedure due to adverse events nor cardiovascular or pulmonary complications. A distorted, broken stent of dimensions 2.5 cm × 1 cm × 1 cm was successfully removed (Fig. 5). Emergence from anesthesia was smooth and rapid after propofol infusion was discontinued.

## 3 Discussion

Bronchoscopy without sedation is a painful and uncomfortable experience.^[17]^ TCI anesthesia with propofol and short-acting opioids is reported to provide satisfactory anesthesia for flexible bronchoscopy, when assisted with repeated topical anesthesia every 10 to 15 minutes.^[18]^ However, cough, apnea, hypoxemia during procedure could not be fully avoided and thus disrupted the procedure.^[17–19,21]^ Previous study reported that anesthesia for flexible bronchoscopy required TCI via propofol Cp of 3 to 4 μg/ml.^[17]^ It has also been demonstrated that effect-site C_50_ concentration of propofol was 2.2 μg/ml when subjects had no response to verbal stimulation and shaking. But to abolish response to painful stimulus, the additional remifentanil effect-site C_50_ 3.3 ng/ml was required.^[20]^ Another study using TCI propofol for fiberoptic intubation demonstrated that mean Cp was 2.8±0.4 μg/ml to achieve non-responsiveness with alfentanil 5 to 10 μg/kg bolus.^[21]^ In this case, the stimulus of rigid bronchoscopy was much stronger than flexible bronchoscopy and fiberoptic intubation. But with assistance of SLN block, the propofol Cp we used was only 1.8 to 2.6 μg/ml, which was lower than the previous studies. Moreover, there were no need for additional opioid infusion and no adverse events, such as cough, apnea and hypoxemia, during the whole procedure. According to the previous report, incidence of hypoxemia during anesthesia for flexible bronchoscopy was 30% to 50% and high cough score was demonstrated.^[17]^

Anesthesia for rigid bronchoscopy usually requires general anesthesia because of the extreme stimulation caused by the rigid metal bronchoscope and working instruments. For lengthier interventions, the aforementioned ventilation techniques may raise concerns of hypoxemia, hypercapnia, and other perioperative complications.^[7–9]^ Comparisons of different ventilation methods are summarized in Table 1. First, apneic oxygenation is an older method that can be applied only for short procedures and puts patients at risk of respiratory acidosis.^[8]^ This technique is now seldomly used. Second, spontaneous assisted ventilation can be applied by titrating an intravenous agent to induce hypnosis while maintaining spontaneous ventilation.^[3]^ Continuous ventilation and oxygenation can be provided throughout the procedure; however, hypoxemia, laryngospasm, and bucking are common complications.^[16]^ Third, controlled ventilation can be applied through an anesthetic circuit connected to the side port of a rigid bronchoscope. Patients are paralyzed, and mechanical ventilation is provided during the procedure. Circuit leakage is a common problem, and hypoxia may be caused by inadequate ventilation and awareness, if anesthesia is maintained using inhaled agents.^[3,8]^ Furthermore, diaphragmatic displacement caused by mechanical ventilation may complicate the intervention. Last, jet ventilation is a widely used technique. Jet ventilation can be provided by a manual jet, low-frequency jet, high-frequency jet, or jet with a combination of ventilation frequencies. Jet ventilation offers an immobile surgical field by paralyzing the patient and provides uninterrupted ventilation during the procedure, but risks of barotrauma and hypercapnia are present.^[4,7,9]^ In addition to these conventional ventilation methods, high-flow nasal cannula has reportedly been increasingly used in flexible bronchoscopy.^[22,23]^ It has also been used during the induction of rigid bronchoscopy to reduce the incidence of desaturation before insertion of the bronchoscope.^[24]^ Nevertheless, oxygen supplementation and ventilation through the nasal route are not feasible during rigid bronchoscopy because the metal working channel of the rigid bronchoscope occupies the entire glottic area and thus limits flow through the vocal cord.

**Table 1:**
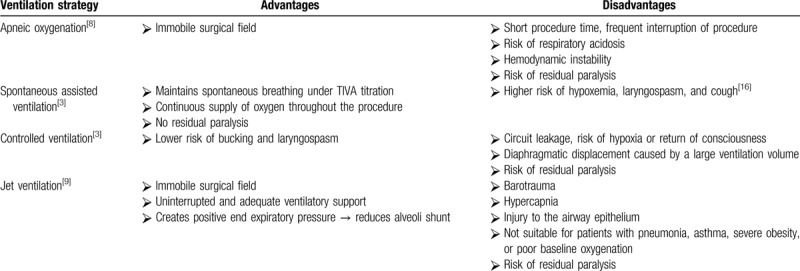
Comparison of ventilation methods.

In consideration of the general weakness and palliative status in this case, TIVA with maintenance of spontaneous assisted ventilation was preferred. We hoped to avoid the use of muscle relaxants. Endotracheal intubation would be necessary while waiting for the full reversal of the muscle relaxant, which would raise concerns associated with prolonged intubation and the requirement of admission to an intensive care unit. We attempted to minimize adverse events by combining regional anesthesia with TIVA. With adequate analgesia of the airway through ultrasound-guided SLN block, airway irritation was mitigated, propofol Cp could be decreased and the use of remifentanil was eliminated.^[25]^ Respiratory depression related to propofol and opioids was reduced, and the patient could retain adequate spontaneous ventilation without hypoxemia. There was no cough, desaturation, and interruption of procedure even under the strong stimulation of rigid bronchoscopy.

Mechanical stimulation of the larynx may induce various unfavorable reflexes during a procedure. The sensory system of the larynx is primarily innervated through the internal branch of the SLN.^[11–13]^ The SLN originates from the vagus nerve at the C1 and C2 levels of the vertebrae and divides into internal and external branches beneath the internal carotid artery at the C2 level.^[26]^ The internal branch runs inferior or lateral to the greater horn of the hyoid with rare variation^[10]^ and provides sensory innervation to the larynx after penetration of the thyrohyoid membrane (Fig. 1). SLN block minimizes the laryngeal reflex and maintains favorable hemodynamic stability during manipulation of the larynx.^[13,14]^ With the injection of 1 to 2 ml of 2% lidocaine inferior and lateral to the greater horn of the hyoid,^[27]^ SLN block can provide effective analgesia for 60 to 180 minutes.^[12]^ This approach is favorable to a local spray of lidocaine along the airway mucosa, which provides incomplete analgesia for only 8 to 14 minutes,^[28]^ SLN block offers superior and longer-lasting analgesic effects. Owing to a distinct adjustment of the internal branch of the superior laryngeal nerve in which it runs mostly within a distance of 10 mm from the greater horn of the hyoid,^[10]^ ultrasound-guided SLN exhibits a high success rate and rare incidence of complications when the greater horn of the hyoid is used as a sonographic landmark.^[10,29]^ Under a transverse view of the hyoid, considering the greater horn rather than the thin SLN as a sonographic landmark is a simpler technique.

In this case of breast cancer with lung metastases and moderately compromised lung function, ultrasound-guided SLN block in combination with TIVA achieved sufficient and long-duration anesthesia for a prolonged therapeutic rigid bronchoscopy. SLN block minimized the requirement of intravenous anesthetics and provided maintenance of adequate spontaneous ventilation for a 2.5-hour uninterrupted procedure without perioperative complications.

## 4 Conclusions

With the rapid development of interventional pulmonology, the application of therapeutic rigid bronchoscopy is expanding, increasing the demand for anesthesia. The administration of anesthesia for rigid bronchoscopy is challenging because the anesthesiologist and the interventionist use the same working channel. For patients for whom there are concerns regarding difficulty in ventilation weaning or who are expected to undergo a prolonged procedure, ultrasound-guided SLN block provides excellent assistance in balanced anesthesia.

## Acknowledgments

This manuscript was edited by Wallace Academic Editing.

## Author contributions

Conception and design of study: Y.C. Liao, W.C. Wu, C.C. Chang, H.C. Tsai

Drafting the manuscript: Y.C. Liao, M. H. Hsieh, H.C. Tsai

Revising the manuscript critically for important intellectual content: Y.C. Liao, W.C. Wu, M. H. Hsieh, C.C. Chang, H.C. Tsai

Approval of the version of the manuscript to be published: Y.C. Liao, W.C. Wu, M. H. Hsieh, C.C. Chang, H.C. Tsai.
